# Bioactive Compounds Targeting Dihydroceramide and Their Therapeutic Potential in Cancer Treatment

**DOI:** 10.3390/cancers17050909

**Published:** 2025-03-06

**Authors:** Yumi Jang

**Affiliations:** 1 Department of Food Science and Nutrition, University of Ulsan, Ulsan 44610, Republic of Korea; yjang@ulsan.ac.kr; Tel.: +82-52-259-2374; 2 Basic-Clinical Convergence Research Institute, University of Ulsan, Ulsan 44610, Republic of Korea

**Keywords:** bioactive compound, cancer, dihydroceramide, sphingolipid

## Abstract

Dihydroceramide (dhCer) was previously considered an inactive precursor of ceramide, a well-known sphingoid base involved in regulating apoptosis and cell death. However, recent studies have shown that dhCer plays a crucial role in various important cellular responses. In this review, we summarize the latest findings on the biological functions of dhCer and the enzymes involved in its biosynthesis. We specifically focus on the emerging evidence implicating dhCer in cancer, as well as its role in regulating key processes such as cell cycle arrest, autophagy, apoptosis, ER stress, and oxidative stress. Furthermore, we discuss bioactive compounds that can modulate dhCer levels in cancer cells, highlighting their potential therapeutic applications in counteracting cancer progression. This review emphasizes the growing recognition of dhCer as a bioactive sphingolipid metabolite with significant potential for cancer therapy.

## Simple Summary

Dihydroceramide (dhCer) was once considered an inactive precursor, but recent studies show that it plays an important role in various cellular processes, including cell death regulation, stress response, and autophagy. It is particularly implicated in cancer, where it may influence the progression of the disease. This review discusses how dhCer affects cancer cells and explores potential therapeutic approaches that target its regulation. By understanding dhCer’s role, researchers are uncovering its potential for developing new cancer treatments and targeting cancer progression more effectively.

## 1. Introduction

**Figure 1:**
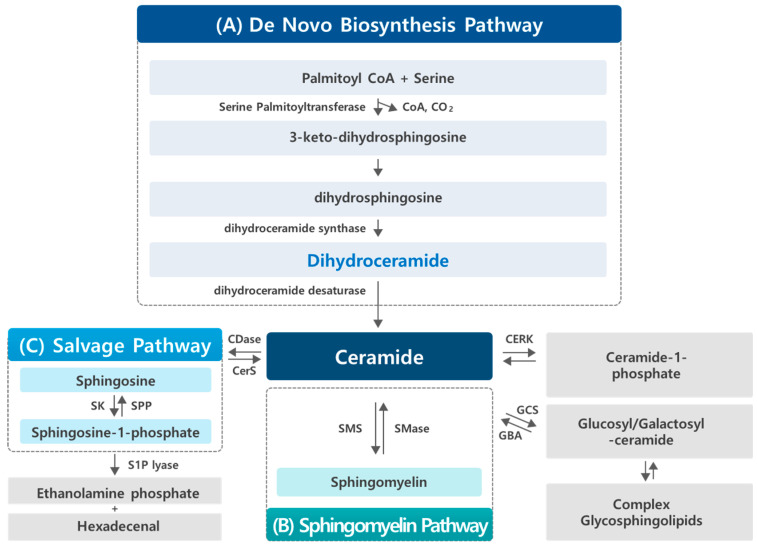
Sphingolipid metabolism pathways. Sphingolipid metabolism occurs through three major pathways: the de novo biosynthesis pathway, the sphingomyelin pathway, and the salvage pathway. (**A**) De novo biosynthesis pathway: this pathway takes place in the endoplasmic reticulum (ER), where the condensation of serine and palmitoyl-CoA by serine palmitoyltransferase (SPT) generates the sphingoid backbone, initiating the synthesis of dhCer. DhCer can then be further desaturated to form Cer, which can be used in the synthesis of complex sphingolipids. (**B**) Sphingomyelin pathway: in the Golgi apparatus, Cer serves as a precursor for the synthesis of SM and other complex sphingolipids via the action of enzymes like sphingomyelin synthase (SMS). The catabolism of complex sphingolipids begins in the lysosome, where SM and other complex sphingolipids are hydrolyzed to release Cer, which is further processed or recycled. (**C**) Salvage pathway: the salvage pathway allows for the recycling of sphingolipid metabolites. Sph, generated during sphingolipid breakdown, can be re-acylated to form dhCer, which can be converted into Cer or other complex sphingolipids. In addition, Sph is phosphorylated by sphingosine kinase (SK) to produce S1P, which can be metabolized or cleared by S1P lyase, linking sphingolipid metabolism to various cellular processes. Key enzymes involved in these pathways include sphingomyelin synthase (SMS), ceramide kinase (CERK), glucosylceramide synthase (GCS), acid glucocerebrosidase (GBA), sphingosine kinase (SK), and S1P phosphatase (SPP).

Sphingolipids are highly bioactive signaling molecules that serve not only as components of cell membranes, but also as regulators of important cellular functions including cell proliferation, differentiation, migration, apoptosis, autophagy, and cell death. Sphingolipid metabolism involves complex pathways that generate diverse sphingolipid species, including de novo biosynthesis, the synthesis of sphingomyelin and glycosphingolipids, and salvage pathways (Figure 1). These routes enable the synthesis of various sphingolipid metabolites, such as ceramide (Cer), sphingosine (Sph), sphingosine-1-phosphate (S1P), and sphingomyelin (SM). Among the various species of sphingolipids, many researchers have focused on Cer, Sph, and S1P for a long time. Cer and Sph are known to inhibit cell proliferation and promote apoptosis. In contrast, S1P, produced through the phosphorylation of Sph by Sph kinase, promotes cell growth and inhibits apoptosis [1,2].

Aberrations in sphingolipid metabolism are closely linked to cancer progression, as dysregulation of key sphingolipid species can lead to altered cell survival, drug resistance, and metastasis. For example, increased levels of S1P have been implicated in tumor growth, angiogenesis, and resistance to chemotherapy, while Cer accumulation has been associated with enhanced apoptotic responses in cancer cells. The balance between Cer and S1P, often referred to as the “sphingolipid rheostat,” plays a critical role in determining cell fate, and disruptions in this balance are commonly observed in various cancers [3,4]. In line with this, Cer has received considerable attention as a potent inducer of apoptosis, while dihydroceramide (dhCer) was previously thought to be an inactive precursor of Cer. However, recent studies have reported that dhCer also affects various biological processes, including cell cycle arrest, autophagy, apoptosis, ER stress, and oxidative stress, and may play an important role in pathophysiological disease states, such as cancer and metabolic diseases [5,6,7]. Emerging evidence suggests that dhCer can modulate cancer cell survival and response to therapy by influencing stress-related signaling pathways. Moreover, certain chemotherapeutic agents, such as fenretinide, have been reported to elevate dhCer levels, leading to enhanced cytotoxicity in cancer cells [8,9].

We will review the evidence that implicates dhCer in various biological processes, with a particular focus on its roles in cancer. Additionally, we will explore various bioactive compounds that can modulate dhCer levels.

## 2. Sphingolipid Metabolism

Sphingolipid metabolism is characterized by its complexity and structural diversity, largely due to the multitude of enzymes involved in their synthesis and degradation. This enzymatic variety enables the generation of a wide range of sphingolipid species. Among these diverse species, dhCer is of particular interest, and we will explore the pathways, enzymes, and precursors that contribute to its synthesis.

### 2.1. Dihydroceramide Generation in the De Novo Synthesis Pathway of Sphingolipid

**Figure 2:**
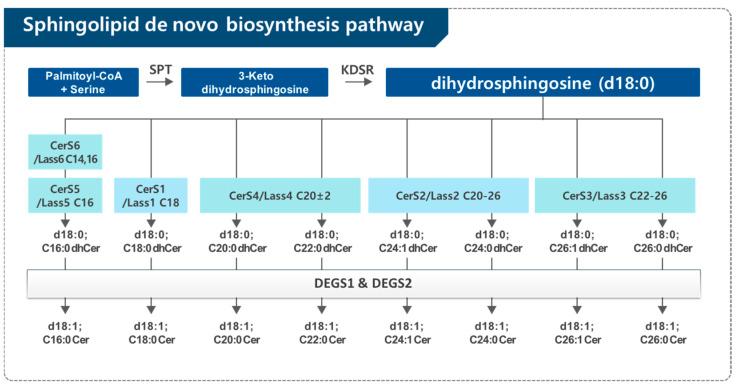
De novo biosynthesis pathway of sphingolipids. The pathway begins with the synthesis of dhSph, which is acylated by ceramide synthases (CerS/Lass) using specific fatty acyl-CoAs. Each dhCer produced can then be desaturated by DEGS to form the corresponding Cer, which can be further converted into more complex sphingolipids. SPT, serine palmitoyltransferase; KDSR, 3-ketodehydrosphingosine reductase; CerS, (dihydro)ceramide synthases; Cer, ceramide; dhCer, dihydroceramide; DEGS, (dihydro)ceramide desaturases.

Sphingolipids are made via the de novo biosynthesis pathway in the endoplasmic reticulum (ER) (Figure 1). Condensation of palmitoyl-CoA and serine forms 3-keto-dihydrosphingosine (sphinganine) by serine palmitoyltransferase (SPT), which is the first enzyme in the de novo sphingolipid biosynthesis pathway. The intermediate 3-keto-dihydrosphingosine is rapidly reduced to dihydrosphingosine (dhSph) by a NADPH-dependent 3-keto-dihydrosphingosine reductase (KDSR). The dhSph is acylated by a family of ceramide synthases (CerSs) using different fatty acyl-CoAs to generate the corresponding dhCer subspecies. In mammals, six genes that encode CerS have been recently cloned. Individual CerS isoforms show substrate preference for specific chain length fatty acyl CoAs, thus generating distinct Cers with distinct acyl-chain lengths ranging from 16 to 26 carbons. Then, the different dhCers are reduced by dihydroceramide desaturase (DEGS) to the final product in the de novo sphingolipid biosynthesis pathway, Cer [10,11,12] (Figure 2).

### 2.2. Dihydroceramide Desaturase (DEGS)

In the de novo sphingolipid biosynthesis pathway, the main enzymes which are directly related to dhCer levels are dhCer synthase and DEGS. In terms of dhCer biosynthesis, dhCer synthase plays a role to convert dhSph to dhCer. Another enzyme that can affect the levels of dhCer is dhCer Δ4-desaturase, which plays a critical role in the final step of Cer synthesis by introducing a 4,5-trans-double bond into dhCer. The first biochemical characterization of the DEGS reaction was performed by Michel et al. (1997) [13] using rat liver microsomes. Their results demonstrate that DEGS employs molecular oxygen as an electron acceptor to add a hydroxyl group at the C4 position of dhSph. This is followed by a NADPH-dependent dehydration reaction, resulting in the formation of a double bond between the C4 and C5 positions of dhCer. DEGS can utilize both NADH and NADPH as electron donors for the reduction of oxygen, generating NAD(P)+. These findings confirmed that the conversion of dhCer to Cer is mediated by a desaturase, not a dehydrogenase [13].

Two isoforms of DEGS have been identified: DEGS1 and DEGS2. Ternes et al. (2002) [14] used a bioinformatics approach to identify a family of sphingolipid Δ4-desaturases, homologous to the Drosophila melanogaster degenerative spermatocyte gene 1, des-1. DEGS1, the human homolog of des-1, exhibits high Δ4-desaturase activity toward dhCer and minimal C-4 hydroxylase activity. On the other hand, DEGS2, an ortholog present in both mice and humans, functions as both a sphingolipid C-4 hydroxylase and a Δ4-desaturase, contributing to the production of either phytoceramide or Cer. Like the other enzymes in the de novo sphingolipid biosynthesis pathway, DEGS is localized in the ER membrane, where it interacts with dhCer species. The activity of DEGS1 is influenced by several factors, such as the alkyl chain length of the sphingoid base (C18 > C12 > C8), fatty acid type (C8 > C18), the stereochemistry of the dhSph moiety (D-erythro-isomer > L- or D-threo-isomers), and the nature of the headgroup (highest activity with dhCer and approximately 20% activity with dhSM). Consequently, research on the role of dhCer frequently uses cell or animal models with DEGS gene knockouts or DEGS inhibitors, which help elevate dhCer levels for further study [14]. DEGS activity is inhibited by several compounds, including cyclopropene-containing ceramide and GT-11 (also known as C8-cyclopropenylceramide; C8-CPPC), which act as competitive inhibitors of DEGS [15,16].

## 3. Findings and Biological Activities of Dihydroceramide

DhCer, a sphingolipid metabolite, has long been considered an inert intermediate in the Cer biosynthesis, primarily serving as a substrate for the enzyme DEGS. The key structural difference between Cer and dhCer is the presence of a 4,5-trans-double bond in Cer, which is absent in dhCer. This subtle distinction makes the differentiation between these two lipids challenging, especially considering that Cer is typically found in concentrations 10- to 100-fold greater than dhCer in most tissues. Initial studies suggested that dhCer lacked significant biological activity. For instance, Bielawska et al. (1993) reported that C2-dihydroceramide (C2-dhCer), a short-chain analog, did not inhibit cell growth or induce apoptosis in HL-60 cells, unlike its counterpart C2-ceramide (C2-Cer) [17]. Similarly, Ahn and Schroeder (2002) found that dhCer, in contrast to sphingosine and C2-Cer, had no effect on cell proliferation or apoptosis in colon cancer HCT116 and HT-29 cell lines [18]. These findings reinforce the notion that dhCer was significantly less bioactive than Cer.

However, these studies primarily utilized short-chain analogs such as C2-dhCer and C2-Cer, which do not fully recapitulate the properties of natural long-chain Cers. As a result, the functional potential of long-chain dhCer remained largely overlooked. Recent technological advances, particularly in lipidomic profiling, have revealed that dhCer may not be as inert as previously thought. With the advent of more precise lipidomic technologies, particularly liquid chromatography–tandem mass spectrometry (LC-MS/MS), the ability to measure dhCer and distinguish it from Cer has provided new insights into the functional roles of dhCer.

Studies using LC-MS/MS have shown that the compound N-(4-hydroxyphenyl)retinamide (4-HPR, or fenretinide), a synthetic retinoid known to induce cell growth arrest and apoptosis in various cancer cell lines, actually accumulates dhCer rather than Cer. This accumulation occurs due to the inhibition of DEGS by fenretinide, which prevents the conversion of dhCer to Cer. This finding challenges the previous assumption that fenretinide elevates Cer levels to mediate its effects on cancer cells and suggests that dhCer may play an active role in mediating cellular responses. These 2006 findings by Zheng and Merrill were a surprising discovery, as they marked the first indication that dhCer, previously considered an inert substance, could have biological activity, thus shifting the perspective on its role in cellular processes [8].

Furthermore, the availability of DEGS inhibitors as pharmacological tools has proven crucial in elucidating the roles of dhCer [19]. The inhibition of DEGS has allowed researchers to distinguish the specific effects of dhCer from Cer and to recognize dhCer as a biologically active lipid with unique functions. Notably, fenretinide has been identified as a direct in vitro inhibitor of DEGS, demonstrating dose- and time-dependent inhibition of the enzyme. This inhibition leads to dhCer accumulation, thereby modulating cellular processes such as autophagy and contributing to the drug’s anticancer effects [20].

Studies involving DEGS1 knockout (KO) cells have demonstrated that the absence of DEGS1 leads to the accumulation of dhCer, which activates both anabolic and catabolic signaling pathways. This results in enhanced autophagy, resistance to apoptosis, and increased cell size, highlighting the complex and multifaceted roles of dhCer in cellular processes [21]. These advancements highlight the importance of conducting “sphingolipidomic” analyses, which involve the comprehensive profiling of sphingolipid species in a given system. By using structure-specific and quantitative measurement techniques such as LC-MS/MS, researchers can now detect and analyze all relevant sphingolipid species, including different forms of Cer and dhCer, in small samples with high precision [22]. As a result of these technological and scientific progressions, the bioactivities associated with dhCer levels that have been identified to date are summarized below.

First, dhCer has been implicated in cellular stress responses, where it regulates autophagy, a critical mechanism for cellular maintenance and survival under stress conditions [8,19]. Specifically, Gagliostro et al. (2012) [19] demonstrated that the accumulation of dhCer, induced by the DEGS inhibitor XM462, triggers autophagy in gastric carcinoma HCG27 cells without causing cell death. This accumulation also affects the cell cycle, delaying the G1/S transition and modulating cyclin D1 expression [19]. Similar effects on cell cycle regulation have been observed in neuroblastoma cells, where the inhibition of DEGS1 led to the accumulation of dhCer and resulted in cell cycle arrest during the G0/G1 phase [23]. Moreover, dhCer influences ER stress pathways in gastric carcinoma cells treated with DEGS inhibitor by activating the translation inhibitor eIF2α and splicing the pro-survival transcription factor Xbp1, contributing to a cellular survival response [19]. Autophagy, while primarily a cell survival mechanism, can also lead to cell death depending on the context. For instance, Hernández-Tiedra et al. (2019) found that ∆^9^-Tetrahydrocannabinol (THC)-induced autophagy in glioma cells leads to cancer cell death through the accumulation of dhCer [24]. Similarly, ABTL0812 has been shown to induce cytotoxic autophagy in cancer cells by increasing dhCer levels [25]. In contrast, resveratrol-induced autophagy in gastric cancer cells also involves a rise in dhCer levels, but with no cell death observed, highlighting a context-dependent role of autophagy in cancer cells [26].

Additionally, dhCer has been shown to play a significant role in oxidative stress. Accumulation of dhCer can contribute to the generation of reactive oxygen species (ROS), which are key mediators of cellular oxidative stress. Recent studies have demonstrated that dhCer induces oxidative stress by promoting the activation of the Rac1-NADPH oxidase complex, leading to increased ROS production and neurodegeneration [27]. On the other hand, oxidative stress itself can also influence dhCer levels. For example, Idkowiak-Baldys et al. (2010) [28] investigated the effects of oxidative stress on DEGS. In their study, cells treated with H_2_O_2_, menadione, or tert-butylhydroperoxide exhibited a significant increase in dhCer levels, while total Cer levels remained relatively unchanged. This increase in dhCer was attributed to the inhibition of DEGS activity, suggesting that oxidative stress impedes the conversion of dhCer to Cer, resulting in dhCer accumulation. Interestingly, no changes in the enzyme’s protein levels were observed, indicating that oxidative stress does not degrade DEGS, but rather inhibits its activity [28]. This suggests that dhCer accumulation can act as both a consequence and a driver of oxidative stress, especially in neurodegenerative conditions. Moreover, previous findings show that under hypoxic conditions, the inhibition of DEGS leads to the accumulation of dhCer, which in turn inhibits cell proliferation, further highlighting the critical role of dhCer in regulating cellular processes in response to environmental stress [29].

In addition to its role in oxidative stress, dhCer has also been implicated in modulating inflammation. Studies have shown that the accumulation of dhCer can regulate anti-inflammatory pathways, such as by upregulating the NF-κB inhibitor A20, thereby attenuating inflammatory responses. This highlights the immunomodulatory potential of dhCer and its relevance to diseases driven by inflammation, such as cancer and neurodegenerative disorders [30,31].

Furthermore, dhCer plays a role in apoptosis by regulating pathways that determine cell survival or death, which may have profound implications for diseases such as cancer [32,33,34]. Studies have shown that dhCer can modulate apoptotic sensitivity by interfering with Cer channel formation, thereby altering apoptotic dynamics [35]. Notably, DEGS knockdown leads to a substantial accumulation of dhCer without affecting Cer levels, which in turn alters apoptotic dynamics by attenuating photodynamic therapy (PDT)-induced late apoptosis and overall cell death while enhancing early apoptosis. These findings suggest that dhCer may regulate apoptotic signaling through mechanisms distinct from those of Cer [36]. Additionally, combining PDT with fenretinide, a DEGS inhibitor, results in increased dhCer levels, which enhances mitochondrial depolarization, caspase-3 activation, and clonogenic cell death, ultimately improving the antitumor efficacy of PDT [37]. Consequently, alterations in dhCer levels or its metabolism have been implicated in a variety of human diseases, underscoring its potential as a therapeutic target for modulating these vital cellular processes [8,21,23].

## 4. Impact of Bioactive Compounds on Dihydroceramide: Focus on Cancer

Recent evidence suggests that dhCer plays a critical role in cancer biology, beyond its traditional characterization as a simple metabolic intermediate. Dysregulation of sphingolipid metabolism, including alterations in dhCer levels, has been implicated in tumor progression, drug resistance, and response to therapeutic interventions. In particular, studies have shown that dhCer accumulation can induce ER stress, trigger autophagy, and modulate apoptosis, all of which are crucial determinants of cancer cell fate. Furthermore, dhCer levels have been found to fluctuate in response to various chemotherapeutic agents, highlighting its potential role as both a biomarker and a therapeutic target in oncology [5,6,7]. Given these findings, understanding the modulation of dhCer by bioactive compounds is of particular interest, as it may offer new avenues for enhancing anticancer therapies. In this section, we summarize the effects of various bioactive compounds that regulate dhCer levels and their potential implications in cancer treatment, as outlined in Table 1.

The first pivotal research on the role of dhCer in cancer emerged in 2004, when Jiang et al. identified γ-tocopherol (γT) as the first natural compound to induce apoptotic cell death in human prostate cancer (LNCaP, PC-3) and lung cancer (A549) cells through the elevation of intracellular dhCer and dhSph, before the widespread use of advanced lipidomic techniques such as LC-MS/MS [32]. This study marked the beginning of a broader interest in dhCer as an active sphingolipid with potential implications for cancer therapy. Prior to this discovery, dhCer was largely considered an inactive precursor in sphingolipid metabolism. However, a lipidomic study conducted by the Merrill group in 2006 revealed that fenretinide, a chemotherapeutic compound, increased dhCer levels in human prostate cancer (DU145) cells, challenging the prior understanding of sphingolipid biology and suggesting that dhCer could play an active role in regulating cellular responses to stress, including autophagy [8]. Expanding on Jiang et al.’s previous work, our study further investigated the role of γ-tocotrienol (γTE) and vitamin E metabolites, 13′-carboxychromanols in promoting cell death in human colon and breast cancer cells. This research demonstrated that vitamin E treatment led to a marked accumulation of dhCer and dhSph, sphingolipid intermediates in the de novo sphingolipid biosynthesis pathway, preceding or coinciding with biochemical and morphological signs of cell death, including apoptosis, necrosis, and autophagy. Notably, of the vitamin E metabolites, 13′-carboxychromanols had the most pronounced effect on dhCer and dhSph accumulation, followed by γTE, while γT exhibited less potent cellular uptake and a correspondingly weaker effect. The accumulation of dhCer and dhSph was critical for vitamin E-induced cell death, as cancer cell viability was significantly reduced in response to these sphingolipid intermediates. In addition, treatment with myriocin, a specific inhibitor of de novo sphingolipid synthesis, partially but significantly counteracted vitamin E-induced autophagy and cell death, underscoring the central role of sphingolipid metabolism in vitamin E’s anticancer activity. However, prolonged vitamin E exposure led to a concomitant increase in Cer levels, attributed to sphingomyelinase activity, which coincided with enhanced cell death. These findings suggest that the elevation of dhCer and dhSph could represent a novel anticancer mechanism, highlighting the therapeutic potential of targeting sphingolipid pathways in cancer treatment [34,40].

The accumulation of dhCer has been increasingly associated with the regulation of autophagy, a crucial cellular process that helps cells manage stress by degrading and recycling cellular components. Several bioactive compounds, including fenretinide [8], resveratrol [26], THC [24], 13′-carboxychromanols [40], γTE [34], and the anticancer drug ABTL0812 [25], have been shown to elevate dhCer levels, triggering autophagy in cancer cells. However, the effects of dhCer accumulation on autophagy depend on the context. While autophagy can promote cancer cell survival in some situations, it can also lead to cell death under conditions of excessive autophagy [19,24]. A pivotal study by Hernández-Tiedra et al. (2016) [24] provided insights into how autophagy can shift from a survival mechanism to a death-inducing process depending on the specific cellular context and molecular signals involved. In this study, the researchers used THC, the primary active component of marijuana, to trigger autophagy-mediated cancer cell death. THC treatment led to a significant increase in the dhCer/Cer ratio in the ER of glioma cells, a critical alteration in sphingolipid metabolism. This shift toward elevated dhCer levels was directed toward autophagosomes and autolysosomes, where it played a central role in promoting lysosomal membrane permeabilization and the subsequent release of cathepsins, which are lysosomal enzymes that contribute to the activation of apoptotic cell death [24]. The key finding of this study was that THC-induced autophagy triggered cytotoxic autophagy, leading to cancer cell death, whereas other autophagic stimuli, such as nutrient deprivation, did not result in this outcome. This distinction is crucial in understanding the conditions under which autophagy leads to cell survival versus when it induces cell death. The increased dhCer levels caused by THC treatment appear to be a critical factor in this process, highlighting the potential for targeting dhCer accumulation as a strategy to selectively induce cancer cell death through autophagy [24]. These findings highlight the dual role of autophagy in cancer therapy and suggest that dhCer accumulation in response to certain treatments, such as THC, may shift the balance toward autophagy-mediated cancer cell death rather than survival. Our group’s work further supports this mechanism, demonstrating that γTE and 13′-carboxychromanols also induce dhCer accumulation, which in turn promotes both autophagy and apoptosis, ultimately contributing to cancer cell death [33,34,40]. Furthermore, these findings highlight the need for a deeper understanding of the molecular pathways that regulate the transition between protective and cytotoxic autophagy in cancer cells.

In addition to autophagy, dhCer accumulation has been shown to activate ER stress and the unfolded protein response (UPR), which attempts to restore cellular homeostasis but, when overstressed, can lead to apoptosis. The UPR, triggered by sustained dhCer accumulation, acts as a checkpoint in cellular decision-making between survival and death signals, further complicating the role of dhCer in cancer cell fate [19]. These complex interactions between dhCer, autophagy, and apoptosis underscore the potential of manipulating dhCer levels for therapeutic purposes in cancer treatment [24,40].

Moreover, dhCer has been implicated in regulating the cell cycle, particularly in inducing a delay in the G1/S phase transition. This delay is important in cancer therapy, as it can interrupt the uncontrolled proliferation of cancer cells. While Gagliostro et al. (2012) [19] suggested that dhCer accumulation might lead to cell cycle arrest and increased autophagy, thereby promoting cancer cell survival, other studies indicate that elevated dhCer levels can also induce cancer cell death. For instance, the work by our group [34,40] and by Munoz-Guordiola et al. (2021) [25] demonstrated that bioactive compounds such as vitamin E metabolites 13′-carboxychromanols, γTE, and the anticancer drug ABTL0812 promote dhCer accumulation, triggering autophagy and ER stress, leading to cancer cell death. Notably, the sphingosine kinase 2 inhibitor ABC294640 has been reported to increase dhCer levels and inhibit prostate cancer cell proliferation, further emphasizing the therapeutic potential of targeting dhCer metabolism in cancer treatment [39].

More recent studies have further explored the potential of modulating dhCer levels for cancer therapy. The anticancer drug ABTL0812, a first-in-class small molecule currently in clinical evaluation in a phase 2 trial in patients with advanced solid tumors, has been found to inhibit DEGS activity, leading to an increase in dhCer levels, the induction of ER stress, and subsequent autophagy and cancer cell death [25]. These findings highlight the therapeutic potential of modulating dhCer levels to induce cancer cell death via multiple mechanisms, including autophagy and ER stress, but the long-term effects, particularly on normal cells, remain to be fully established.

In conclusion, modulating dhCer levels through bioactive compounds has significant implications for cellular processes critical to cancer progression. By regulating autophagy, ER stress, apoptosis, and the cell cycle, these compounds can influence cancer cell survival and treatment response. However, to maximize therapeutic efficacy while minimizing adverse effects, further research into the dose-dependent effects and long-term safety profiles of dhCer-modulating compounds is essential. Ongoing research into the molecular mechanisms underlying these processes will provide valuable insights into the therapeutic potential of dhCer-modulating compounds in cancer therapy.

## 5. Conclusions

DhCer is an important bioactive sphingolipid that plays a critical role in cellular processes such as autophagy, cell cycle regulation, ER stress, and apoptosis. Recent advances in lipidomic research and the development of pharmacological inhibitors and transgenic models have unveiled numerous potential therapeutic targets, including specific enzymes involved in dhCer metabolism. This growing body of knowledge suggests that further research into dhCer modulation may yield compounds with significant clinical benefits, particularly for cancer therapy. By identifying compounds that can effectively regulate dhCer levels and its associated pathways, we may be able to target cancer cell death more precisely and enhance treatment outcomes. Ultimately, the continued exploration of dhCer’s role in cancer biology and the identification of bioactive compounds that modulate its activity hold great promise for the development of novel therapeutic strategies. With its potential to effectively treat cancer, dhCer modulation is poised to become a key component of future cancer treatment regimens.

## Figures and Tables

**Table 1:** Bioactive compounds regulating dhCer levels and their effects on cancer cell processes.

Year and Author	Type of Cancer	Treatment	Sphingolipid Levels	Mechanisms/ Results
2004; Jiang et al. [32]	Human prostate (LNCaP, PC-3) and lung (A549) cancer cells	Vitamin E; γ-tocopherol: 50 μM, 1~3 days	dhCer ↑ dhSph ↑	Apoptosis induction/cell death
2006; Zheng et al. [8]	Human prostate cancer cells (DU145)	Fenretinide (4-HPR): 10 μM, 24 h	dhCer ↑	Autophagy induction
2008; Wang et al. [9]	Human cancer cells (MCF-7/AdrR, HL-60, HT-29)	Fenretinide (4-HPR): 10 μM, 24 h	dhCer ↑, dhSph ↑	De novo sphingolipid synthesis ↑ DEGS inhibition
2009; Signorelli et al. [26]	Human gastric epithelial cancer cells (HGC-27)	Resveratrol: 50 μM, 16 h (DEGS inhibitor; XM462)	dhCer (C_14:0, 16:0, 18:0, 20:0, 22:0, 24:0, 24:1_-dhCer) ↑ (Cer levels increased only slightly)	Inhibition of DEGS/ autophagy induction (no sign of cell death)
2009; Schiffmann et al. [38]	Human colon (HCT116, HCA-7, HT-29), cervix (HeLa), lung (A549), breast (MDA-MB231, MCF-7), embryonic kidney (HEK293) cancer cells	Celecoxib: 20, 40, 60, 80 μM, 2 h (Methyl celecoxib 80 μM)	dhCer (C_16:0, 24:0, 24:1_-dhCer) ↑ dhSph ↑ Cer (C_24:0, 24:1_-Cer) ↓	De novo sphingolipid synthesis ↑, DEGS inhibition/anti-proliferation
2012; Gopalan et al. [33]	Human breast cancer cells (MCF-7)	Vitamin E; γ-tocopherol (40 μM) and γ-tocotrienol (10 μM): 2~3 days	dhCer ↑ Cer ↑	JNK/CHOP/DR5-mediated apoptosis induction
2012; Gagliostro et al. [19]	Human gastric cancer cells (HCG27)	DEGS inhibitor; XM462): 8 μM, 8~24 h	dhCer ↑	ER stress, autophagy ↑/delayed cell cycle G1/S transition
2013; Venant et al. [39]	Murine castration-resistant prostate cancer cells (TRAMP-C2)	ABC294640; sphingosine kinase 2 inhibitor: 10~30 μmol/L, 72 h	dhCer ↑	Inhibition of DEGS activity/reduction in cancer cell growth
2016; Jang et al. [40]	Human colon cancer cells (HCT116)	Vitamin E metabolite; 13′-carboxychromanols: 10, 20 μM, 1~16 h	dhCer (C_16:0, 24:0, 24:1_-dhCer) ↑ dhSph ↑ Cer (C_24:0, 24:1_-Cer) ↓	Inhibition of DEGS activity/apoptosis and autophagy induction
2016; Hernàndez-Tiedra et al. [24]	U87MG MEFs cancer cells	THC (∆^9^-Tetrahydrocannabinol; a component of marijuana): 6 μM, 6 h	dhCer (C_16:0, 24:0, 24:1_-dhCer) ↑ Cer ↓	Autophagy-mediated cancer cell death
2017; Jang et al. [34]	Human colon (HCT116), breast (MCF-7) cancer cells	Vitamin E; γ-tocotrienol: 20 μM, 8, 16, 24 h	dhCer, dhSph ↑ Cer 8, 16 h ↓ SM ↓,	Inhibition of DEGS activity/early apoptosis and autophagy induction
2021; Munoz-Guordiola et al. [25]	Human pancreatic (MiaPaca-2) and lung (A549) cancer cells	ABTL0812; anticancer drug: 40~100 μM, 1~24 h	dhCer ↑	Impaired DEGS1 activity/ ER stress-mediated cytotoxic autophagy induction

Abbreviations: Cer, ceramide; dhCer, dihydroceramide; dhSph, dihydrosphingosine; DEGS, (dihydro)ceramide desaturases; ↑, increase; ↓, decrease.

## Data Availability

Not applicable.
